# Supplementary material for: EPS Production by Lacticaseibacillus casei Using Glycerol, Glucose, and Molasses as Carbon Sources
Source: Microorganisms. 2024 Jun 6;12(6):1159. doi: 10.3390/microorganisms12061159 (PMC11205391; doi:10.3390/microorganisms12061159)
Supplement: Supplementary file 1 [file microorganisms-12-01159-s001.zip › microorganisms-2901849-supplementary.pdf]

Supplementary material

Table S1. Microorganisms used for study of EPS production.

	Isolated Strains	Isolation Location	Identification
1	LMISM6	Waste decantation pond from cassava flour industry – Indústria Plaza, located in the region of Sta. Maria-SP	*Lactobacillus plantarum*
2	CH17		*Lactobacillus* sp.
3	CH24	Sauerkraut	*Weissella paramesenteroides*
4	CH25		*Leuconostoc mesenteroides*
5	CCC3		*Pediococcus pentosaceus*
6	CCC15		*Leuconostoc lactis*
7	CC10	Sugar cane juice	*Weissella paramesenteroides*
8	CC28		*Weissella paramesenteroides*
9	CC29		*Weissella paramesenteroides*
10	Ke2		*Lacticaseibacillus casei*
11	Ke7		*Lacticaseibacillus casei*
12	Ke8	Kefir	*Lacticaseibacillus casei*
13	Ke11		*Lacticaseibacillus casei*
14	Ke16		*Pediococcus pentosaceus*
15	BSLM9	Fresh sugar cane bagace from the Santa Lucia mill, located in the region of Araras,SP	*Pediococcus pentosaceus*
16	BSLM14		*Leuconostoc lactis*
17	Y15C	Fermented milk	*Lactobacillus delbrueckii* subsp. *delbrueckii*
18	B512	State University of Campinas (School of Food Engineering)	*Leuconostoc mesenteroides*
19	B103	Provided by professor Georgina Lourdes Michelena Alvarez (Instituto Cubano de Investigaciones de la Caña de Azúcar, CIDCA)	*L. rhamnosus*
20	ALE3	Rosemary rhizosphere	*Bacillus coagulans*
21	ALE4		*Bacillus coagulans*
22	Vini6	Butterbur (*Cenchrus echinatus*)	*Sporolactobacillus nakayamae subsp. nakayamae*
